# Diagnosis of Intrauterine Pregnancy Despite the Presence of Etonogestrel Implant

**DOI:** 10.7759/cureus.28041

**Published:** 2022-08-15

**Authors:** Ryan J Sawyers, Pratik M Parikh, Jose J Lazaro, Marna R Greenberg

**Affiliations:** 1 Department of Emergency and Hospital Medicine, Lehigh Valley Health Network/University of South Florida (USF) Morsani College of Medicine, Allentown, USA; 2 Department of Obstetrics and Gynecology, Lehigh Valley Health Network/University of South Florida (USF) Morsani College of Medicine, Allentown, USA

**Keywords:** anchoring bias, pregnancy detection, contraception, larc, nexaplanon

## Abstract

While contraception is an important method to avoid pregnancy, it is not always effective. Our case details a 33-year-old-female with an etonogestrel implant who presented to the emergency department (ED) with a two-week history of vomiting and abdominal pain. Pelvic and transvaginal ultrasound confirmed a single, live intrauterine pregnancy. Our case serves as a reminder that ED providers should have a high index of suspicion for pregnancy in clinically relevant scenarios, despite contraceptive methods, until the appropriate confirmatory diagnostic evaluation for pregnancy is completed.

## Introduction

Detection of pregnancy in emergency departments (EDs) is critical in preventing complications of ectopic pregnancies and may additionally avert the use of contraindicated diagnostics and therapeutic management in females of childbearing potential. The early presentation of pregnancy includes amenorrhea, nausea with or without vomiting, breast enlargement, increased urinary frequency, and fatigue [1]. Less common early presenting symptoms include uterine cramping, abdominal bloating, constipation, and lightheadedness [1]. Of women presenting with abdominal pain or vaginal bleeding who have a positive pregnancy test, 10% deny the possibility of pregnancy at the time of presentation [2].

The incidence of ectopic pregnancy for the general population is 2%, but this incidence increases to 13%-16% among women of the reproductive age presenting to EDs [3,4]. Ectopic pregnancies account for 3%-5% of all pregnancy-related deaths [5]. Due to ED providers using bedside ultrasound in the evaluation of patients more frequently, practitioners see this increase substantiated and validates that the best practice is to perform an ultrasound on a patient regardless of beta-human chorionic gonadotropin (b-hCG) levels if symptoms suggest an ectopic pregnancy [6]. As testing is readily available in the ED, such as point-of-care pregnancy tests and/or beside pelvic and transabdominal ultrasound, all women of childbearing age should be assumed pregnant until proven otherwise [7].

The use of contraception should not obscure decision-making to diagnose pregnancy in an ED setting. From 2015 to 2017, the Centers for Disease Control (CDC) reported that 65% of women aged 18-49 years used a method of contraception. The leading form of contraception was female sterilization by tubal ligation at 18.3%, followed by oral contraceptives (OCPs) at 12.6%, and long-acting reversible contraception (LARCs), such as the etonogestrel arm implant or intrauterine devices (IUD), at 10.3% [8]. Though their effectiveness for preventing pregnancy is high, each method has reported failures in preventing conception. The CDC reported a failure rate of 0.5% for female sterilization, 7% for OCPs, 0.1% for the etonogestrel implant, 0.1%-0.4% for the levonorgestrel IUD, and 0.8% for the copper IUD [9]. While exceedingly rare, the authors present a case of a 33-year-old female with an etonogestrel implant who presented to the ED and was found to be pregnant.

## Case presentation

A 33-year-old gravida 2, para 2 female presented to the ED with a two-week history of vomiting and generalized abdominal pain. Her past medical history included adenomyosis, irregular menses, gastroesophageal reflux disease treated with daily omeprazole, and constipation treated with lactulose as needed. She had not been able to tolerate any fluids or solids by mouth since symptom onset, and her last bowel movement was five days prior to presentation. She also complained of a lack of appetite and nausea. She did not report dysuria, increased urinary frequency, fever, shortness of breath, or recent infection. She had no known sick contacts, recent travel, or recent antibiotic use.

Her last menstrual period was eight weeks prior to her ED visit. She was scheduled to have a total abdominal hysterectomy with bilateral salpingo-oophorectomy due to a sustained history of chronic pelvic pain that same week but ultimately decided to decline the procedure on the day scheduled. She instead opted for an etonogestrel contraceptive implant after testing negative to a urine pregnancy test seven weeks and four days prior to the ED visit. Her etonogestrel implant insertion occurred nine weeks after curettage for abnormal bleeding in which her pregnancy test was negative, and she was advised to avoid vaginal intercourse. The utilization of interim birth control methodology is not known, nor if she had unprotected sex. The implant procedure occurred without incident, and the implant was inserted correctly per standardized guidelines.

**Figure 1:**
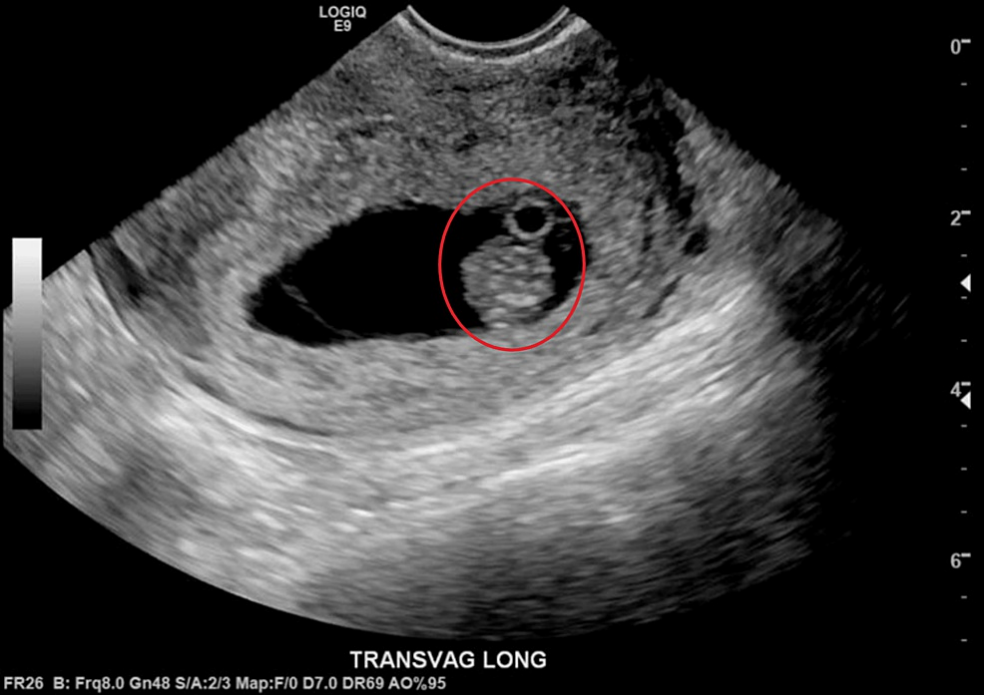
Transvaginal ultrasound showing the longitudinal view of uterus revealing intrauterine gestational sac with single fetal pole and yolk sac

Her presenting vital signs included a blood pressure of 115/76 mm/Hg, pulse of 105 beats per minute, temperature of 98.8°F, respiratory rate of 16 breaths per minute, and oxygen saturation of 98%. She was well-appearing and in no acute distress on the physical exam. She was tachycardic without murmur, and her lungs were clear to auscultation. Her abdominal exam was notable for generalized abdominal tenderness to palpation, yet soft, nondistended, and with normal bowel sounds. She additionally had intact distal pulses without lower extremity edema. A point-of-care urinalysis resulted in a specific gravity greater than 1.030 (reference range: 1.003-1.030), with trace ketones, protein, and leukocytes. Her complete blood count was within normal limits, and the comprehensive metabolic panel was notable for lipase of 43 U/L (reference range: 80-360), but otherwise within normal limits. Her point-of-care urine human chorionic gonadotropin (HCG) was unexpectedly positive, which led the clinician to do a bedside point-of-care pelvic ultrasound in the context of her abdominal pain. The exam showed an early intrauterine pregnancy. Subsequently, her serum b-HCG was 241,025 mIU/mL (reference range: <4). A pelvic ultrasound and transvaginal ultrasound were then performed by the radiology department to formally confirm the results of the HCG test and ensure that the fetus was intrauterine rather than ectopic, given her previous medical history of reproductive tract comorbidities. The ultrasound confirmed a single, live intrauterine pregnancy, with a fetal heart rate of 178 bpm, and an estimated gestational age of eight weeks five days by crown rump length (Figure 1).

She was discharged from the ED with a scheduled follow-up for prenatal care with her obstetrician and had her implant removed two days later. The patient continued to have well visits during the pregnancy and was at 28 weeks of gestation at the time of this report.

## Discussion

In order to maintain the highest quality of care possible, it is important to remain conscious of common cognitive biases when making a diagnosis: whether it is confirmation bias, the selective interpretation of information to conform to one’s own predetermined beliefs; the affect heuristic, the alteration of a practitioner’s decisions based on their emotional reaction to a person; or, as is most relevant to this report, the anchoring bias [10]. Anchoring bias is similar to confirmation bias and involves the prioritization of certain information to support one’s initial impressions, regardless of their validity [10]. These biases are typically unintentional, which is why it is of the utmost importance for practitioners to remain cognizant of them in order to avoid allowing their biases to make their diagnostic decisions for them.

It may seem obvious, but this case provides support for the need of continued pregnancy screening despite factors that may decrease the chance of pregnancy. It is a long-held tenet of emergency medicine to perform a pregnancy screening in patients and to consider women of childbearing age pregnant until proven otherwise. Urine pregnancy tests, b-HCG serum testing, and diagnostic bedside ultrasound are all useful in the diagnosis of pregnancy in an unsuspecting patient and more broadly essential for ruling out ectopic pregnancy [4]. While this standard of care is recognized, there are many barriers in the ED setting that may prevent diagnosis. The inability of a patient to provide a urine sample on demand, the time it takes to get a blood sample collected and analyzed, and the availability of equipment like an ultrasound machine at all EDs may be the factors that prevent diagnosis.

In this case, the recency of the patient’s etonogestrel implant insertion leaves some potential for a false-negative urine pregnancy test before the placement of the implant. If that was true, a false negative may have been the result of HCG levels being too low to detect, as is the case in very early pregnancy. Typically, detection limits of urine HCG tests range from 25 to 200 mIU/mL [11]. This means that pregnancy is usually not detectable until the first missed menstrual period. Since the patient was not experiencing any symptoms that are abnormal for those affected by adenomyosis, there were no contraindications for implant contraception. This additional potential, pregnancy in the face of a potentially false-negative urine test, adds to the case for being exceptionally liberal about considering pregnancy as a part of a differential and, just as importantly, choosing a test with the appropriate sensitivity. Perhaps, using a serum quantitative pregnancy test before procedures might be considered. This only reinforces the necessity of carefully selected tools for the exclusion of pregnancy.

In addition to her clinical symptoms, the patient was particularly sure she could not be pregnant. Her confidence could have allowed her to be resistant to testing or ultimately even could have caused the physician to miss the possible diagnosis. Putting too much credence on this initial conviction from the patient that she “could not be pregnant” would have been consistent with “anchoring bias” in which the clinician’s ability to objectively take in further information is distorted by this initial perception [12]. Diagnostic errors are common among clinicians who succumb to anchoring effects [13-16]. Even a delay in diagnosis can be harmful, especially in the context of the increasing utilization of computerized tomography in the ED setting [17]. A readily available computed tomography technician may take a patient to imaging before pregnancy testing is resulted. Leading to more complexity since, while the imaging may have been necessary, counseling and obtaining informed consent for imaging that involves radiation would certainly expect to be different if the pregnancy was involved [18].

Specific to our case, while removing or stopping contraception when a positive pregnancy test is resulted is generally advised, if removed in early pregnancy, the complications are severely lessened [19]. Significant literature is not available for adverse events in pregnancy related to etonogestrel implants likely due to the low prevalence of failure as a contraceptive agent. In the context of complications described from other forms, such as the IUD (higher miscarriage, preterm labor incidence, and chorioamnionitis), it seems likely that the best outcomes are associated with early detection and removal of the implant [19].

## Conclusions

Pregnancy is possible in those patients using contraceptive implants. Though the etonogestrel implant is highly effective at preventing pregnancy, one should not anchor this part of the patient’s history as rule-out criteria for intra- or extra-uterine pregnancy. Cognitive bias rooted from their presence, or contraception of any kind, should not alter the threshold for emergency clinicians to utilize diagnostic screening in women of childbearing potential. While exceedingly rare, our case serves as a reminder that even those with an etonogestrel implant may be pregnant.
